# Allelic frequency differences of *DAOA* variants between Caucasians and Asians and their association with major mood disorders

**DOI:** 10.1038/s41392-019-0066-5

**Published:** 2019-10-04

**Authors:** Zhihua Yang, Shuang Zhang, Fuquan Zhang, Yao Yao, Kwangwoo Kim, David Meyre, Hongmei Zhang, Hai Liao, Shuquan Rao, Xinhe Huang

**Affiliations:** 1 School of Life Science and Engineering, Southwest Jiaotong University, Chengdu, 610031 China; 2 Hospital of Chengdu University of Traditional Chinese Medicine, Chengdu, 610072 China; 3 Wuxi Mental Health Center, Nanjing Medical University, Wuxi, 214151 China; 4 Institute of Basic Medical Sciences, Chinese Academy of Medical Sciences & Peking Union Medical College, Beijing, 10005 China; 5 Department of Biology, Kyung Hee University, Seoul, 02447 Korea; 6 Department of Health Research Methods, Evidence, and Impact, McMaster University, Hamilton, ON L8S 4K1 Canada; 7 Department of Pathology and Molecular Medicine, McMaster University, Hamilton, ON L8S 4K1 Canada; 8 Department of Biology, Georgia State University, Atlanta, GA 30303 USA

**Keywords:** Neurodevelopmental disorders, Molecular biology

**Dear Editor,**

Major mood disorders, which primarily include bipolar disorder (BD) and major depressive disorder (MDD), are among the most common psychiatric disorders and are recognized as leading causes of morbidity worldwide. Family, twin and adoption studies have consistently indicated moderate-to-strong genetic contributions to the risk of major mood disorders.^1^ Genetic association and genome-wide association studies (GWAS) suggest that there is some degree of overlap for some specific disorders but also specific genetic diversity.^2^ *DAOA* (D-amino acid oxidase activator), a gene located on human chromosome 13q33.2, plays a crucial role in the central nervous system through binding with *DAO* (encoding D-amino acid oxidase). Convergent lines of biological evidence suggest that *DAOA* is an attractive candidate gene for major mood disorders.^2,3^ Multiple studies have been conducted to characterize the association of *DAOA* with major mood disorders in diverse populations, but inconsistent results have been reported.^4^ The inconsistent associations of SNPs in *DAOA* with major mood disorders may be explained by various study assertions, ethnic heterogeneity, and insufficient statistical power. Here, we collected all available genetic and phenotypic data from diverse samples (including GWASs and candidate gene studies) to perform a systematic meta-analysis of seven SNPs across the *DAOA* gene locus (65,087 subjects and 1022 nuclear families) and further investigate the functional consequence of risk SNP rs2391191 on *cis*-regulation of *DAOA* expression and binding affinity to transcription factor TCF4.

Genetic association studies of the *DAOA* gene with major mood disorders have primarily focused on the following seven single-nucleotide polymorphisms (SNPs): rs2391191, rs3918342, rs1421292, rs3916965, rs778294, rs947267, and rs1935062. The genomic information of the seven SNPs is summarized elsewhere (the online version of this article, which contains supplementary materials). In total, our meta-analysis included 27 independent samples consisting of 29,003 cases of mood disorder and 36,084 healthy subjects, as well as 1022 nuclear families. Meta-analysis using the fixed-effect model showed that rs2391191 (risk allele: A) was significantly associated with major mood disorders in both Caucasian (*P* = 4.32E-04, OR = 0.952, 95% CI = 0.926–0.978) and overall samples (*P* = 1.79E-04, OR = 0.957, 95% CI = 0.934–0.980), but not in Asian populations (*P* = 0.308, OR = 0.975, 95% CI = 0.928–1.024). Similar analyses were performed for rs3916965 (risk allele: A), rs3918342 (risk allele: T), rs1421292 (risk allele: A), rs778294 (risk allele: A), rs947267 (risk allele: C), and rs1935062 (risk allele: C). Our meta-analyses revealed no significant association between each of the six SNPs and BD, MDD or major mood disorders in any population (Caucasian, Asian or combined) (all *P* > 0.05). More detailed descriptions of meta-analytic results for each SNP can be found in the online version of this article, which contains supplementary materials.

As expected, the frequency of the risk allele (A) of rs2391191 differed dramatically between Caucasian and Asian populations. In detail, the A allele appeared to be the minor allele in Caucasian populations, but the major allele in Asian populations. More interestingly, rs2391191 was fixed for the ancestral allele (G allele) in some African populations. Furthermore, an obvious difference in A allele frequency of rs2391191 was observed between Caucasian and Asian populations (see the online version of this article, which contains supplementary materials). Collectively, the dramatic differences in allelic frequency strongly suggested that rs2391191 might have experienced recent positive selection in human populations, thus leading to differential association with major mood disorders in different populations.

Given that rs2391191 is located in a potential enhancer region, we conducted functional analysis of this SNP. Brief bioinformatics analysis using RegulomeDB (http://www.regulomedb.org/) revealed that this locus is located in a putative binding site of the common transcription factor TCF4 (Fig. 1a). The change from G to A at rs2391191 may influence the binding affinity of TCF4, resulting in different *DAOA* expression levels (Fig. 1b). To verify the functional consequences of rs2391191, we performed an electrophoretic mobility shift assay (EMSA) with nuclear protein extracts from 293T cells and labeled double-stranded oligo probes containing either the rs2391191 A allele or G allele. As shown in Fig. 1c, the predicted binding sequence containing the G allele had a higher binding affinity than that containing the A allele. In addition, the shifted band was significantly abolished by 100x or 200x excess unlabeled probes (Fig. 1c). These findings suggested that TCF4 might preferentially bind to the G allele rather than the A allele, consistent with the previous prediction.

**Fig. 1:**
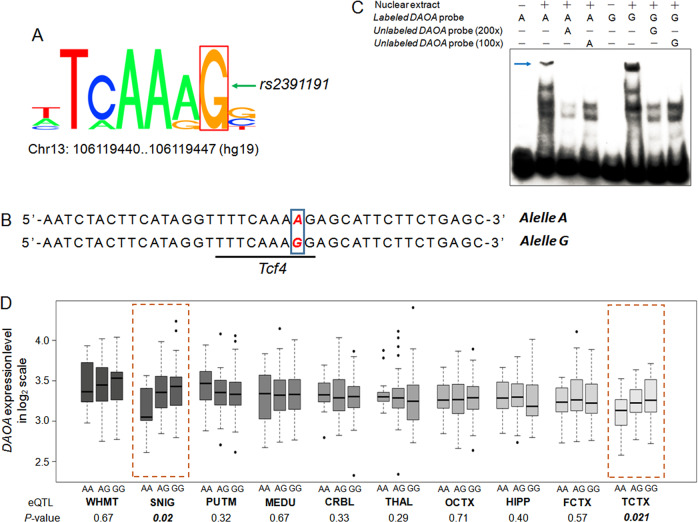
Risk allele of rs2391191 alters the binding affinity of the transcription factor *TCF4* and predicts *DAOA* mRNA expression. **a** Schematic diagram of the transcription factor *TCF4-*binding motif (from RegulomeDB, http://www.regulomedb.org/). **b** Genomic sequence, covering the binding motif, for testing the binding activity of the transcription factor TCF4. Alleles of rs2391191 are highlighted in red. **c** EMSA data using the nuclear protein extracts of 293T cells transiently transfected with pcDNA3.1-*TCF4*. Lanes 1 and 5: negative control; lanes 2 and 6: the probes containing the G allele and the A allele can both bind TCF4, but the binding affinity of the G allele is stronger than that of the A allele; lanes 3–4 and 7–8: 100-fold and 200-fold molar excess of unlabeled probes with the G allele or A allele were introduced, independently. Representative EMSA from one intact gel is presented here. **d** Expression of *DAOA* significantly differed by genotypes of rs2391191 in the human substantia nigra (SNIG) and temporal cortex (TCTX). The data were extracted from BRAINEAC (http://www.braineac.org/). WHMT intralobular white matter, PUTM putamen (at the level of the anterior commissure), MEDU inferior olivary nucleus (subdissected from the medulla), CRBL cerebellar cortex, THAL thalamus (at the level of the lateral geniculate nucleus), OCTX occipital cortex, HIPP hippocampus, FCTX frontal cortex

To validate the effects of rs2391191 on *DAOA* gene expression in vivo, we checked BrainEAC, a well-characterized expression database based on ten distinct brain regions from 134 European individuals free of neurodegenerative disorders.^5^ Among the ten brain regions investigated, rs2391191 was significantly associated with *DAOA* expression (Affymetrix ID: t3524289) in both the substantia nigra (SNIG, *P* = 0.020) and temporal cortex (TCTX, *P* = 0.021), with the risk allele A having lower expression (Fig. 1d), suggesting tissue-specific regulation of rs2391191 on *DAOA* expression. Taken together, our data support a *cis*-regulatory effect on *DAOA* expression by which disease-associated SNPs underpin the pathogenesis of major mood disorders. However, further studies are necessary to investigate whether the expression levels of *DAOA* are changed in subjects with major mood disorders compared with the levels in healthy subjects.

In summary, we found a promising SNP, rs2391191, showing significant association with major mood disorders in Caucasian populations, but not in Asian populations. The ORs for rs2391191 in Caucasian populations (for BD: 0.966; for MDD: 0.949; for major mood disorders: 0.952) are comparable with those of other risk genes for major mood disorders reported in previous large-scale meta-analyses.^6^ Moreover, the functional assay demonstrated that rs2391191 can influence transcription factor TCF4-binding affinity and predict *DAOA* expression in the substantia nigra and temporal cortex. Our findings confirmed that *DAOA* is a risk gene for major mood disorders and might provide a better understanding of the potential biological mechanism underlying major mood disorders.

There are, however, limitations to the interpretation of our results. First, the sample size is still insufficient to reach a more definitive conclusion, and we are not able to include some recent samples from the GWAS data, because the statistical results are not available yet.^7,8^ Second, our study only focused on seven SNPs in/near the *DAOA* gene, and further studies should focus on other *DAOA* SNPs, even rare variants, to evaluate the association between *DAOA* variation and major mood disorders. Third, stratified analysis based on gender, disease onset age, comorbidity, impairment in brain morphology, and subtypes of disease could not be performed in this study due to insufficient information. Finally, *DAOA* may exert its function by interacting with other genetic risk components, which calls for conjoint analysis with other candidate genes.

## Supplementary information

Supplementary Materials Word.

Supplementary Materials PDF.

Table S1 Excel

Table S1 PDF
