# Supplementary material for: Allelic frequency differences of DAOA variants between Caucasians and Asians and their association with major mood disorders
Source: Signal Transduct Target Ther. 2019 Oct 4;4:39. doi: 10.1038/s41392-019-0066-5 (PMC6799844; doi:10.1038/s41392-019-0066-5)
Supplement: Supplementary file 1 — Supplementary Materials Word. [file 41392_2019_66_MOESM1_ESM.doc]

Supplementary Materials for

**Allelic frequency differences of *DAOA* variants between Caucasians and Asians and their association with major mood disorders**

Zhihua Yang^1^,·Shuang Zhang^2^, Fuquan Zhang^3^,·Yao Yao^4^,· Kwangwoo Kim^5^,·David Meyre^6,7^,·Hongmei Zhang^8^,·Hai Liao^1^, Shuquan Rao^1,*^,·Xinhe Huang^1,*^

^1^School of Life Science and Engineering, Southwest Jiaotong University, Chengdu, 610031, China·

^2^Hospital of Chengdu University of Traditional Chinese Medicine, Chengdu, 610072, China·

^3^Wuxi Mental Health Center, Nanjing Medical University, Wuxi, 214151, China

^4^Institute of Basic Medical Sciences, Chinese Academy of Medical Sciences & Peking Union Medical College, Beijing, 10005, China

^5^Department of Biology, Kyung Hee University, Seoul 02447, Korea

^6^Department of Health Research Methods, Evidence, and Impact, McMaster University, Hamilton, ON L8S 4K1, Canada

^7^Department of Pathology and Molecular Medicine, McMaster University, Hamilton, ON L8S 4K1, Canada

^8^Department of Biology, Georgia State University, Atlanta, GA 30303, USA

^*^These authors jointly directed this work.

Correspondence to: Dr. Shuquan Rao ([Raosq@swjtu.edu.cn](mailto:Raosq@swjtu.edu.cn)) and Dr. Xinhe Huang ([Xinhehuang@swjtu.edu.cn](mailto:Xinhehuang@swjtu.edu.cn))

**This PDF file includes:**

Materials and Methods

Supplementary Text

Figures. S1 to S10

Tables. S1 to S14

**Materials and methods**

**Search strategy**
The search strategy was designed to identify all sources of published data both in the literatures and in available databases. Systematic literature search was conducted for eligible studies from PubMed (http://www.ncbi.nlm.nih.gov), SCOPUS (http://www.scopus.com), EMBASE (<http://www.elsevier.com/online-tools/embase>), ISI Web of Knowledge (<http://apps.webofknowledge.com/>), and Google scholar (<https://scholar.google.com/>). The following combined searching terms were employed: “*DAOA*”, “D-amino-acid oxidase activator”, “G72”, “polymorphism or SNP”, “bipolar disorder”, “manic depressive disorder”, “major depressive disorder” and “unipolar disorder”. The last searching was undertaken on April 10, 2017. The references of retrieved articles were manually checked to identify other eligible studies. The authors were contacted if full articles or genotypic data could not be accessed.

Besides, three large-scale GWASs for BD or MDD conducted by Psychiatric Genomics Consortium (PGC) [1, 2] or CONVERGE consortium[3] were also included in the present meta-analyses. The detailed information of sample ascertainment, diagnosis, genotyping quality control, and statistical analyses in both of these studies, can be found in the original publications. In order to exclude any possibility of sample overlap between candidate studies and GWASs, we carefully checked the sample description, including ethnicity, regions, and sample size, in each study. If samples overlapped with those from GWASs, the study was therefore excluded from the current meta-analysis.

**Eligibility criteria and data extraction**
The included studies were consistent with the following criteria: 1) articles published in peer-reviewed English journals; 2) case-control studies (population-based or family-based); 3) case status being defined of major mood disorders (BD or MDD), with control subjects having no history of mental disorder, other neurological

disorders, alcohol dependence, or drug dependence; 4) genotype frequencies in the controls were in Hardy-Weinberg equilibrium (HWE) (*P* > 0.05); and 5) samples were independent among different studies (if samples from different studies overlapped, only the studies with the largest sample size were included).

The following information was collected from each included study using a standard data extraction form by two independent researchers (SR and XH): 1) first author; 2) publication year; 3) sample origins and ethnicities; 4) sample characteristics, *i.e.*, sample size, age and gender distribution; 5) diagnostic criteria of cases; 6) genotyping method; 7) necessary data of each SNP for meta-analyses, *i.e.*, allele or genotype counts, and odds ratio (OR) with standard error (SE) or 95% confidence interval (95% CI).

**Statistical analyses**

For meta-analysis, OR and SE from each study were used to calculate the pooled OR and 95% CI. OR and SE were calculated using the following two alternative methods if they were absent in recruited studies. For case-control designs, the OR and SE were analyzed through RevMan (<http://tech.cochrane.org/revman>) if total genotype/allele counts of each group were available; for family-based samples, the OR of transmission disequilibrium test (TDT) and associated SE were calculated using the following formula:

$$\text{OR}=\frac{\text{a}}{\text{b}}$$

$$\text{SE}=\text{SQRT}(\frac{1}{a}+\frac{1}{b})$$

where “a” means the number of transmitted A allele from heterozygous parents to affected offspring, while “b” indicates the number of not transmitted A allele.

Before meta-analyses, publication bias was examined using the Begg’s funnel plot test of asymmetry and represented as a funnel plot. Briefly, in the absence of publication bias, one could expect to see that the majority of studies may fall inside the pseudo-confidence region with bounds OR ± 1.96 SE, thus forming a funnel shape. The heterogeneity between studies was assessed by Cochran's

chi-square-based Q statistic and the inconsistency index (*I^2^*). The fixed-effect model was used to combine data in the absence of between-study heterogeneity; otherwise, the random-effect model was applied. Subgroup analysis was further examined according to ethnicity (Caucasian and Asian). We used a forest plot to graphically present the pooled OR and the 95% CI. Each study was represented by a square in the plot, the size of which corresponded to the weight of each study. All the analyses were accomplished with the Stata Statistical Package (Version 12.0).

**Electrophoretic mobility shift assay (EMSA)**

To express human Tcf4 proteins in mammalian cells, full-length human *TCF4* cDNA was subcloned into pcDNA3.1 plasmid (pcDNA3.1-*TCF4*). Nuclear extraction proteins of 293T cells over-expressing *TCF4* were extracted strictly according to NE-PER nuclear and cytoplasmic extraction reagents (Pierce, USA). Biotin-labeled oligonucleotides at the 3’-end and unlabeled oligonucleotides, containing rs2391191 were synthesized in Invitrogen. The nucleotide sequences of the double-stranded oligonucleotides with either A or G allele were:

A allele: 5’- AATCTACTTCATAGGTTTTCAAA***A***GAGCATTCTTCTGAGC-3’;

G allele: 5’- AATCTACTTCATAGGTTTTCAAA***G***GAGCATTCTTCTGAGC-3’.

After annealing of two complementary oligonucleotides, EMSA was performed using Light Shift Chemiluminescent EMSA Kit (pierce, USA) strictly according to the manufacture’s protocol. For competition experiments, 100-fold and 200-fold molar excess of unlabeled double stranded oligonucleotides were added to the binding reaction.

**Supplementary Text**

**Significant association of rs2391191 (risk allele: A) with major mood disorders in Caucasian populations**

Genetic association studies of *DAOA* gene with major mood disorders have primarily focused on the following 7 single nucleotide polymorphisms (SNPs): rs2391191, rs3918342, rs1421292, rs3916965, rs778294, rs947267 and rs1935062. The genomic information of the 7 SNPs was summarized in **Fig. S1**. **Table S1** listed the information of each study, *i.e.*, sample origin, sample size, age and gender distribution, criteria of definition, etc (if available). **Table S2** listed the information of the studies involving rs2391191 in this meta-analysis and the association results in each individual sample. Assessment of publication bias was performed for BD and MDD studies, respectively. As shown in **Fig. S2**, no potential publication bias was found for either BD (*P* = 0.586) or MDD (*P* = 0.322) groups as evidenced by the Begg’s funnel plot test of asymmetry. For BD, subgroup analyses by ethnicity using the fixed-effect model revealed significant association of rs2391191 in Caucasian populations (*P* = 0.040, OR = 0.961, 95% CI = 0.924-0.998), but no association in Asian populations (*P* = 0.502) **(Fig. S3A)**. Similarly, significant association of rs2391191 with MDD was observed both in the Caucasian (*P* = 0.012, OR = 0.949, 95% CI = 0.912-0.989) and overall samples (P = 0.006, OR = 0.956, 95% CI = 0.926-0.987), but not in Asian populations (*P* = 0.198) **(Fig. S3B)**.

**Meta-analyses of the remaining 6 SNPs with major mood disorders**

***rs3916965 (risk allele: A)***

Meta-analyses of the remaining 6 SNPs with major mood disorders are detailed in **Tables S3-S14** and **Figs S4-S9** as follows.

rs3916965 is a G to A substitution, which is located 5’ upstream of the *DAOA* gene locus. Totally, we identified 11 independent case-control or family samples from 10 studies of either BD[4-9] or MDD[3, 10-12]. Overall, the BD studies included 2,173 cases and 2,996 controls, as well as 376 families, and the MDD studies included 6,611 cases and 6,997 controls **(Table S3)**. As indicated by the Begg’s funnel plot test of asymmetry, there was no potential publication bias for either the BD (*P* = 0.293) or MDD (*P* = 1.000) samples **(Fig. S2)**. Since no obvious between-study heterogeneity was observed for BD or MDD and each subgroup according to ethnicity (all *P* > 0.05), the fixed-effect model was applied to combine data from individual studies. Meta-analyses showed no association of rs3916965 with either BD or MDD (both *P* > 0.05). Moreover, we divided the samples according to ethnicity, and performed subgroup meta-analysis. Again, no positive association was found for BD in either Caucasian or Asian populations (*P* > 0.05); however, a trend of association of rs3916965 with MDD was observed in Caucasian populations (*P* = 0.053, OR = 0.873, 95% CI = 0.761-1.002). Furthermore, we combine BD and MDD samples together, and found no association of this locus in either Caucasian, Asian or overall populations (all *P* > 0.05) **(Table S9)**.

***rs3918342 (risk allele: T)***

rs3918342 is a C to T substitution, which is located 3’ downstream of the *DAOA* gene locus. We found 19 case-control or family sample from 16 studies that associated rs3918342 with BD or MDD[3-18]. Since overlapped samples were found between Schumacher *et al.*[7] and Schulze *et al.*[8] study, we therefore excluded Schulze *et al.* study in this part of analyses. Characteristics of each eligible study involving rs3918342 were listed in **Table S4**, and **Table S10** showed the results of meta-analyses for BD, MDD and major mood disorders. In total, the BD studies included 10,180 patients of BD, 12,959 controls and 376 families; while the MDD studies enrolled 15,908 cases and 16,704 controls. No evidence of publication bias was observed for either BD (*P* = 0.547) or MDD (*P* = 0.589) samples **(Fig. S3)**.

Meta-analyses using the fixed-effect model, due to absence of between-study heterogeneity, showed that there was no significant association between rs3918342 and either BD or MDD (both *P* > 0.05). Subgroup analyses according to ethnicity also revealed no sign of association of rs3918342 in either population for both BD and MDD (all *P* > 0.05). When the BD and MDD samples were combined together as a single group major mood disorders, meta-analysis still showed non-significant association **(Table S7)**.

***rs1421292 (risk allele: A)***

rs1421292, a T to A substitution, is also located 3’ downstream of the *DAOA* gene locus. In summary, 13 studies consisting of 14 independent samples of major mood disorders were recruited in this meta-analysis **(Table S5)**[3-8, 10-13, 16, 17, 19]. The overall samples included 10,220 BD patients, 376 BD families, 6,611 MDD patients and 20,594 healthy subjects. Based on the Begg’s funnel plot test of asymmetry, we found no evidence of publication bias for either BD or MDD samples, as indicted by funnel plots (both *P* >0.05) **(Fig. S4)**.

Using the fixed-effect model, we found no significant association of this locus with either BD (*P* = 0.461) or MDD (*P* = 0.643). We also divided BD or MDD according to ethnicity, or combine them together as major mood disorders; again, meta-analyses revealed no sign of association in either group **(Table S11)**.

***rs778294 (risk allele: A)***

rs778294, located in DAOA exon 6, is a synonymous variant (NM_001161812.1, c.495 G>A). A total of 8 independent samples from 7 studies, which consisted of 10005 cases, 12721 controls and 376 BD families, were identified for BD[5-7, 9, 13, 17, 19] **(Table S6)**. No potential publication bias was observed using the Begg’s funnel plot test of asymmetry (*P* = 0.458) **(Fig. S5)**. We found marginal association of this locus with BD in Asian populations (475 cases and 588 controls, *P* = 0.047, OR = 0.047, 95% CI = 0.587-0.997); while in Caucasian and overall samples, our samples detected no sign of association (both *P* > 0.05) using the fixed-effect model **(Table S12)**. For MDD, 4 studies including a total of 30837 samples (15351 cases and 15486 controls) were enrolled in the meta-analysis and the results exhibited no significant association of rs778294 with either Asian (the random-effect model were applied), Caucasian or overall populations (the random-effect model were applied) (all *P* > 0.05). Combined analysis of BD and MDD also produced negative association of rs778294 **(Table S12)**.

***rs947267 (risk allele: C)***

rs947267 is located in DAOA intron 6 with a A to C substitution. A total of 11 samples of rs947267 were identified, including 7 BD samples from 6 studies (8911 cases, 11223 controls and 376 BD families)[4, 5, 13, 14, 17, 20], 3 samples of MDD (5848 cases and 6005 controls)[3, 12, 14], and one mixed sample of major mood disorders (646 families) **(Table S7)**. There was no potential publication bias for either BD (*P* = 0.938) or MDD (*P* = 0.821) studies, as indicated by the Begg’s funnel plot test of asymmetry **(Fig. S6)**. As shown in **Table S13**, our samples revealed no significant association of rs947267 with either BD (*P* = 0.513) or MDD (P = 0.549). According to ethnicity, we divided both BD and MDD samples into Caucasian and Asian populations, respectively; again, no association was observed for either subgroup. We further combined BD and MDD samples together and found no association between rs947267 and major mood disorders. All meta-analyses were performed using the fixed-effect model due to absence of between-study heterogeneity **(Table S13)**.

***rs1935062 (risk allele: C)***

rs947267 is also located in intron 6 of the DAOA gene locus. Totally, our BD samples included 2216 cases, 3453 controls and 376 families from 7 studies[4-7, 14, 20, 21], and our MDD samples enrolled 5360 cases and 5525 controls from 2 studies[3, 14] **(Table S8)**. Notably, no potential publication bias was observed for either the BD or MDD studies **(Fig. S7).** Our meta-analyses revealed no significant association of this locus with BD, MDD, major mood disorders in any population (Caucasian, Asian or combined) (all *P* > 0.05), using the fixed-effect model since no heterogeneity was detected among studies **(Table S14)**.

**Table S2 Characteristics of included studies of *DAOA* rs2391191 in BD and MDD**

| **Author, year** | **Risk allele** | **Region** | **Ethnicity** | ***N* cases** | ***N* controls** | **OR** | **95% CI** |
| --- | --- | --- | --- | --- | --- | --- | --- |
| **BD** | | | | | | | |
| Chen, 2004 | A | USA | Caucasian | 139 | 113 | 0.811 | 0.566-1.161 |
| Schumacher, 2004 | A | Germany | Caucasian | 300 | 300 | 0.880 | 0.696-1.111 |
| Williams, 2006 | A | U.K. | Caucasian | 706 | 1416 | 0.889 | 0.779-1.014 |
| Maheshwari, 2008 | A | Europe | Caucasian | 543 | 549 | 1.079 | 0.908-1.283 |
| Maheshwari, 2008 | A | Europe | Caucasian | 376 families | | 1.262 | 0.948-1.680 |
| Bass, 2009 | A | U.K. | Caucasian | 303 | 433 | 1.060 | 0.853-1.316 |
| Gawlik, 2010 | A | Germany | Caucasian | 191 | 188 | 0.895 | 0.695-1.216 |
| Gaysina, 2010 | A | Canada | Caucasian | 385 | 312 | 0.959 | 0.765-1.202 |
| Gaysina, 2010 | A | U.K. | Caucasian | 515 | 1316 | 1.088 | 0.925-1.279 |
| Grigoroiu-Serbanescu, 2010 | A | Romania | Caucasian | 198 | 180 | 0.924 | 0.690-1.239 |
| Soronen, 2011 | A | Finland | Caucasian | 178 | 1322 | 0.849 | 0.677-1.065 |
| PGC-BD, 2012 | A | Multiple | Caucasian | 7481 | 9250 | 0.955 | 0.908-1.005 |
| Hukic, 2013 | A | Sweden | Caucasian | 488 | 1044 | 0.940 | 0.796-1.11 |
| Zhang, 2009 | A | China | Asian | 475 | 588 | 1.120 | 0.933-1.344 |
| Chiesa, 2012 | A | Korea | Asian | 132 | 170 | 0.869 | 0.623-1.214 |
| **MDD** | | | | | | | |
| Rietschel, 2008 | A | Germany | Caucasian | 500 | 1030 | 0.874 | 0.748-1.021 |
| Gawlik, 2010 | A | Germany | Caucasian | 57 | 188 | 1.277 | 0.828-1.970 |
| Soronen, 2011 | A | Finland | Caucasian | 272 | 1322 | 0.846 | 0.701-1.022 |
| PGC-MDD, 2013 | A | Multiple | Caucasian | 9240 | 9519 | 0.958 | 0.943-1.029 |
| Arias, 2014 | A | Spain | Caucasian | 320 | 150 | 0.980 | 0.735-1.316 |
| Chen, 2012 | A | China | Asian | 488 | 480 | 0.787 | 0.657-0.943 |
| Chiesa, 2012 | A | Korea | Asian | 145 | 170 | 1.038 | 0.747-1.445 |
| CONVERGE-MDD, 2015 | A | China | Asian | 5303 | 5337 | 0.983 | 0.931-1.038 |
| **Major mood disorders*^1^*** |  |  |  |  |  |  |  |
| Gomez, 2008 | A | Hungary | Caucasian | 646 families | | 0.820 | 0.689-0.975 |

^1^Major mood disorders, mixed BD and MDD samples.

**Table S3 Characteristics of included studies of *DAOA* rs3916965 in BD and MDD**

| **Author, year** | **Risk allele** | **Region** | **Ethnicity** | ***N* cases** | ***N* controls** | **OR** | **95% CI** |
| --- | --- | --- | --- | --- | --- | --- | --- |
| **BD** | | | | | | |  |
| Schumacher, 2004 | A | Germany | Caucasian | 300 | 300 | 1.137 | 0.900-1.436 |
| Schulze, 2005 | A | Germany | Caucasian | 300 | 300 | 1.163 | 0.680-1.099 |
| Maheshwari, 2008 | A | Europe | Caucasian | 376 families | | 1.244 | 0.930-1.664 |
| Gaysina, 2010 | A | U.K. | Caucasian | 515 | 1316 | 1.088 | 0.924-1.281 |
| Gaysina, 2010 | A | Canada | Caucasian | 385 | 312 | 0.959 | 0.765-1.202 |
| Grigoroiu-Serbanescu, 2010 | A | Romania | Caucasian | 198 | 180 | 0.924 | 0.690-1.239 |
| Zhang, 2009 | A | China | Asian | 475 | 588 | 0.893 | 0.743-1.073 |
| **MDD** | | | | | | |  |
| Rietschel, 2008 | A | Germany | Caucasian | 500 | 1030 | 0.845 | 0.723-0.987 |
| Arias, 2014 | A | Spain | Caucasian | 320 | 150 | 0.980 | 0.730-1.310 |
| Chen, 2012 | A | China | Asian | 488 | 480 | 0.891 | 0.744-1.067 |
| CONVERGE-MDD, 2015 | A | China | Asian | 5303 | 5337 | 0.986 | 0.933-1.042 |

**Table S4 Characteristics of included studies of *DAOA* rs3918342 in BD and MDD**

| **Author, year** | **Risk allele** | **Region** | **Ethnicity** | ***N* cases** | ***N* controls** | **OR** | **95% CI** |
| --- | --- | --- | --- | --- | --- | --- | --- |
| **BD** |  |  |  |  |  |  |  |
| Schumacher, 2004 | T | Germany | Caucasian | 300 | 300 | 0.755 | 0.602-0.948 |
| Schulze, 2005 | T | Poland | Caucasian | 294 | 311 | 0.917 | 0.719-1.163 |
| Maheshwari, 2008 | T | Europe | Caucasian | 376 families | | 0.990 | 0.745-1.314 |
| Bass, 2009 | T | U.K. | Caucasian | 303 | 433 | 1.246 | 1.012-1.535 |
| Zuliani, 2009 | T | U.K. | Caucasian | 38 | 81 | 1.321 | 0.765-2.283 |
| Gawlik, 2010 | T | Germany | Caucasian | 191 | 188 | 1.235 | 0.928-1.642 |
| Gaysina, 2010 | T | U.K. | Caucasian | 515 | 1316 | 1.083 | 0.923-1.271 |
| Gaysina, 2010 | T | Canada | Caucasian | 385 | 312 | 0.961 | 0.770-1.199 |
| Grigoroiu-Serbanescu, 2010 | T | Romania | Caucasian | 198 | 180 | 1.066 | 0.801-1.419 |
| PGC-BD, 2012 | T | Multiple | Caucasian | 7481 | 9250 | 1.028 | 0.982-1.076 |
| Zhang, 2009 | T | China | Asian | 475 | 588 | 0.923 | 0.776-1.098 |
| **MDD** |  |  |  |  |  |  |  |
| Rietschel, 2008 | T | Germany | Caucasian | 500 | 1030 | 0.880 | 0.756-1.024 |
| Gawlik, 2010 | T | Germany | Caucasian | 57 | 188 | 1.066 | 0.701-1.621 |
| PGC-MDD, 2013 | T | Multiple | Caucasian | 9240 | 9519 | 1.003 | 0.962-1.046 |
| Arias, 2014 | T | Spain | Caucasian | 320 | 150 | 0.930 | 0.710-1.230 |
| Chen, 2012 | T | China | Asian | 488 | 480 | 1.057 | 0.884-1.264 |
| CONVERGE-MDD, 2015 | T | China | Asian | 5303 | 5337 | 1.017 | 0.963-1.074 |
| **Major mood disorders** |  |  |  |  |  |  |  |
| Gomez, 2008 | T | Hungary | Caucasian | 646 families | | 0.910 | 0.768-1.079 |

**Table S5 Characteristics of included studies of *DAOA* rs1421292 in BD and MDD**

| **Author, year** | **Risk allele** | **Region** | **Ethnicity** | ***N* cases** | ***N* controls** | **OR** | **95% CI** |
| --- | --- | --- | --- | --- | --- | --- | --- |
| **BD** | | | | | | | |
| Schumacher, 2004 | A | Germany | Caucasian | 300 | 300 | 1.222 | 0.974-1.534 |
| Schulze, 2005 | A | Poland | Caucasian | 294 | 311 | 1.000 | 0.787-1.25 |
| Williams, 2006 | A | Multiple | Caucasian | 706 | 1416 | 0.965 | 0.849-1.096 |
| Maheshwari, 2008 | A | Europe | Caucasian | 376 families | | 0.990 | 0.745-1.314 |
| Bass, 2009 | A | U.K. | Caucasian | 303 | 431 | 0.850 | 0.691-1.047 |
| Zuliani, 2009 | A | U.K. | Caucasian | 38 | 81 | 0.844 | 0.487-1.465 |
| Gaysina, 2010 | A | U.K. | Caucasian | 515 | 1316 | 1.041 | 0.888-1.221 |
| Gaysina, 2010 | A | Canada | Caucasian | 385 | 312 | 1.000 | 0.802-1.247 |
| Grigoroiu-Serbanescu, 2010 | A | Romania | Caucasian | 198 | 180 | 1.015 | 0.763-1.351 |
| PGC-BD, 2012 | A | Multiple | Caucasian | 7481 | 9250 | 1.024 | 0.977-1.073 |
| **MDD** | | | | | | | |
| Rietschel, 2008 | A | Germany | Caucasian | 500 | 1030 | 1.156 | 0.993-1.335 |
| Arias, 2014 | A | Spain | Caucasian | 320 | 150 | 0.787 | 0.599-1.042 |
| Chen, 2012 | A | China | Asian | 488 | 480 | 0.937 | 0.781-1.125 |
| CONVERGE-MDD, 2015 | A | China | Asian | 5303 | 5337 | 1.010 | 0.956-1.067 |

**Table S6 Characteristics of included studies of *DAOA* rs778294 in BD and MDD**

| **Author, year** | **Risk allele** | **Region** | **Ethnicity** | ***N* cases** | ***N* controls** | **OR** | **95% CI** |
| --- | --- | --- | --- | --- | --- | --- | --- |
| **BD** | | | | | | | |
| Schumacher, 2004 | A | Germany | Caucasian | 300 | 300 | 0.866 | 0.675-1.110 |
| Williams, 2006 | A | Multiple | Caucasian | 706 | 1416 | 1.075 | 0.935-1.236 |
| Maheshwari, 2008 | A | Europe | Caucasian | 543 | 549 | 0.811 | 0.672-0.978 |
| Maheshwari, 2008 | A | Europe | Caucasian | 376 families | | 0.789 | 0.578-1.077 |
| Bass, 2009 | A | U.K. | Caucasian | 302 | 438 | 1.022 | 0.810-1.290 |
| Grigoroiu-Serbanescu, 2010 | A | Romania | Caucasian | 198 | 180 | 1.057 | 0.771-1.449 |
| PGC-BD, 2012 | A | Multiple | Caucasian | 7481 | 9250 | 0.998 | 0.949-1.050 |
| Zhang, 2009 | A | China | Asian | 475 | 588 | 0.765 | 0.587-0.997 |
| **MDD** | | | | | | | |
| PGC-MDD, 2013 | A | Multiple | Caucasian | 9240 | 9519 | 1.029 | 0.982-1.078 |
| Arias, 2014 | A | Spain | Caucasian | 320 | 150 | 1.050 | 0.770-1.440 |
| Chen, 2012 | A | China | Asian | 488 | 480 | 1.371 | 1.050-1.790 |
| CONVERGE-MDD, 2015 | A | China | Asian | 5303 | 5337 | 0.990 | 0.914-1.073 |

**Table S7 Characteristics of included studies of *DAOA* rs947267 in BD and MDD**

| **Author, year** | **Risk allele** | **Region** | **Ethnicity** | ***N* cases** | ***N* controls** | **OR** | **95% CI** |
| --- | --- | --- | --- | --- | --- | --- | --- |
| **BD** | | | | | | | |
| Chen, 2004 | C | America | Caucasian | 139 | 113 | 1.441 | 1.01-2.058 |
| Maheshwari, 2008 | C | Europe | Caucasian | 376 trios |  | 1.192 | 0.882-1.611 |
| Bass, 2009 | C | U.K. | Caucasian | 303 | 433 | 0.937 | 0.759-1.157 |
| Gaysina, 2010 | C | U.K. | Caucasian | 440 | 955 | 0.960 | 0.816-1.129 |
| Gaysina, 2010 | C | Canada | Caucasian | 357 | 284 | 0.960 | 0.769-1.199 |
| Gawlik, 2010 | C | Germany | Caucasian | 191 | 188 | 0.901 | 0.672-1.209 |
| PGC-BD, 2012 | C | Multiple | Caucasian | 7481 | 9250 | 1.018 | 0.972-1.067 |
| **MDD** |  |  |  |  |  |  |  |
| Gawlik, 2010 | C | Germany | Caucasian | 57 | 188 | 1.188 | 0.776-1.816 |
| CONVERGE-MDD, 2015 | C | China | Asian | 5303 | 5337 | 1.013 | 0.959-1.07 |
| Chen, 2012 | C | China | Asian | 488 | 480 | 1.021 | 0.85-1.225 |
| **Major mood disorders** | | | | | | | |
| Gomez, 2008 | C | Hungary | Caucasian | 646 families | | 1.089 | 0.916-1.294 |

**Table S8 Characteristics of included studies of *DAOA* rs1935062 in BD and MDD**

| **Author, year** | **Risk allele** | **Region** | **Ethnicity** | ***N* cases** | ***N* controls** | **OR** | **95% CI** |
| --- | --- | --- | --- | --- | --- | --- | --- |
| **BD** | | | | | | | |
| Schumacher, 2004 | C | Germany | Caucasian | 300 | 300 | 1.046 | 0.823-1.330 |
| Chen, 2004 | C | America | Caucasian | 139 | 113 | 0.592 | 0.410-0.855 |
| Maheshwari, 2008 | C | Europe | Caucasian | 376 trios | | 1.122 | 0.833-1.511 |
| Gawlik, 2010 | C | Germany | Caucasian | 191 | 188 | 0.985 | 0.736-1.319 |
| Gaysina, 2010 | C | U.K. | Caucasian | 515 | 1316 | 1.138 | 0.963-1.345 |
| Gaysina, 2010 | C | Canada | Caucasian | 385 | 312 | 0.957 | 0.758-1.209 |
| Grigoroiu-Serbanescu, 2010 | C | Romania | Caucasian | 198 | 180 | 1.039 | 0.775-1.393 |
| Hukic, 2013 | C | Sweden | Caucasian | 488 | 1044 | 0.947 | 0.799-1.122 |
| **MDD** | | | | | | | |
| Gawlik, 2010 | C | Germany | Caucasian | 57 | 188 | 1.247 | 0.817-1.904 |
| CONVERGE-MDD, 2015 | C | China | Asian | 5303 | 5337 | 0.982 | 0.930-1.037 |

**Table S9 Statistics of meta-analyses for *rs3916965* with BD, MDD and major mood disorders**

| **Disorder** | **Risk allele** | ***N* of comparisons** | ***N* of case/control,**  ***N* of families** | **Meta-analysis** | | | | **Heterogeneity** | |
| --- | --- | --- | --- | --- | --- | --- | --- | --- | --- |
|  |  |  |  | **OR** | **95% CI** | ***Z*-value** | ***P*-value** | ***I^2^*** | ***P*-value** |
| **BD** |  |  |  |  |  |  |  |  |  |
| Caucasian | A | 6 | 1698/2408, 376 | 1.080 | 0.985-1.185 | 1.63 | 0.103 | 0.0% | 0.605 |
| Asian | A | 1 | 475/588 | 0.893 | 0.743-1.073 | 1.21 | 0.227 | N.A. | N.A. |
| Overall | A | 7 | 2173/2996, 376 | 1.039 | 0.957-1.129 | 0.91 | 0.361 | 13.1% | 0.330 |
| **MDD** |  |  |  |  |  |  |  |  |  |
| **Caucasian** | **A** | **2** | **820/1180** | **0.873** | **0761-1.002** | **1.94** | **0.053** | **0.0%** | **0.380** |
| Asian | A | 2 | 5791/5817 | 0.978 | 0.928-1.030 | 0.85 | 0.398 | 9.3% | 0.294 |
| Overall | A | 4 | 6611/6997 | 0.964 | 0.917-1.012 | 1.48 | 0.139 | 27.6% | 0.247 |
| **Major mood disorders** |  |  |  |  |  |  |  |  |  |
| Caucasian | A | 8 | 2518/3588, 376 | 1.011 | 0.936-1.091 | 0.27 | 0.788 | 34.7% | 0.151 |
| Asian | A | 3 | 6266/6405 | 0.971 | 0.923-1.021 | 1.14 | 0.252 | 0.0% | 0.374 |
| Overall | A | 11 | 8784/9993, 376 | 0.983 | 0.942-1.025 | 0.81 | 0.418 | 25.5% | 0.201 |

**Table S10 Statistics of meta-analyses for *rs3918342* with BD, MDD and major mood disorders**

| **Disorder** | **Risk allele** | ***N* of comparisons** | ***N* of case/control,**  ***N* of families** | **Meta-analysis** | | | | **Heterogeneity** | |
| --- | --- | --- | --- | --- | --- | --- | --- | --- | --- |
|  |  |  |  | **OR** | **95% CI** | **Z-value** | **P-value** | **I^2^** | **P-value** |
| **BD** | | | | | | | | | |
| Caucasian | T | 10 | 9705/12371, 376 | 1.028 | 0.989-1.070 | 1.39 | 0.165 | 38.1% | 0.105 |
| Asian | T | 1 | 475/588 | 0.923 | 0.776-1.098 | 0.90 | 0.366 | N.A. | N.A. |
| Overall | T | 11 | 10180/12959, 376 | 1.023 | 0.984-1.063 | 1.15 | 0.249 | 37.3% | 0.101 |
| **MDD** | | | | | | | | | |
| Caucasian | T | 4 | 10117/10887 | 0.993 | 0.955-1.033 | 0.35 | 0.729 | 0.9% | 0.387 |
| Asian | T | 2 | 5791/5817 | 1.020 | 0.968-1.075 | 0.75 | 0.451 | 0.0% | 0.685 |
| Overall | T | 6 | 15908/16704 | 1.003 | 0.972-1.035 | 0.18 | 0.860 | 0.0% | 0.571 |
| **Major mood disorders** | | | | | | | | | |
| Caucasian | T | 15^1^ | 19822/23258, 1022 | 1.008 | 0.980-1.036 | 0.55 | 0.582 | 31.6% | 0.116 |
| Asian | T | 3 | 6266/6405 | 1.012 | 0.962-1.064 | 0.46 | 0.646 | 0.0% | 0.512 |
| Overall | T | 18 | 26088/29663, 1022 | 1.009 | 0.985-1.033 | 0.70 | 0.481 | 22.2% | 0.191 |

^1^In Gomez *et al.* study, MDD and BPD samples were combined together as “major mood disorders” group [17].

**Table S11 Statistics of meta-analyses for *rs1421292* with BD, MDD and major mood disorders**

| **Disorder** | **Risk allele** | ***N* of comparisons** | ***N* of case/control,**  ***N* of families** | **Meta-analysis** | | | | **Heterogeneity** | |
| --- | --- | --- | --- | --- | --- | --- | --- | --- | --- |
|  |  |  |  | **OR** | **95% CI** | ***Z*-value** | ***P*-value** | ***I^2^*** | ***P*-value** |
| **BD** | | | | | | | | | |
| Caucasian | A | 10 | 10220/13597, 376 | 1.015 | 0.976-1.055 | 0.74 | 0.461 | 0.0% | 0.671 |
| **MDD** | | | | | | | | | |
| Caucasian | A | 2 | 820/1180 | 0.972 | 0.668-1.414 | 0.15 | 0.882 | 82.6% | 0.016 |
| Asian | A | 2 | 5791/5817 | 1.004 | 0.952-1.058 | 0.14 | 0.889 | 0.0% | 0.440 |
| Overall | A | 4 | 6611/6997 | 1.012 | 0.963-1.062 | 0.46 | 0.643 | 56.9% | 0.073 |
| **Major mood disorders** | | | | | | | | | |
| Caucasian | A | 12 | 11040/14777, 376 | 1.018 | 0.981-1.057 | 0.96 | 0.336 | 14.4% | 0.303 |
| Asian | A | 2 | 5791/5817 | 1.004 | 0.952-1.058 | 0.14 | 0.889 | 0.0% | 0.440 |
| Overall | A | 14 | 16831/20594, 376 | 1.013 | 0.983-1.045 | 0.87 | 0.386 | 4.7% | 0.400 |

**Table S12 Statistics of meta-analyses for rs778294 with BD, MDD and major mood disorders**

| **Disorder** | **Risk allele** | ***N* of comparisons** | ***N* of case/control,**  ***N* of families** | **Meta-analysis** | | | | **Heterogeneity** | |
| --- | --- | --- | --- | --- | --- | --- | --- | --- | --- |
|  |  |  |  | **OR** | **95% CI** | ***Z*-value** | ***P*-value** | ***I^2^*** | ***P*-value** |
| **BD** | | | | | | | | | |
| Caucasian | A | 7 | 9530/12133, 376 | 0.987 | 0.946-1.031 | 0.57 | 0.567 | 34.6% | 0.164 |
| **Asian** | **A** | **1** | **475/588** | **0.765** | **0.587-0.997** | **1.98** | **0.047** | **N.A.** | **N.A.** |
| Overall | A | 8 | 10005, 12721, 376 | 0.981 | 0.940-1.024 | 0.89 | 0.376 | 44.7% | 0.081 |
| **MDD** | | | | | | | | | |
| Caucasian | A | 2 | 9560/9669 | 1.029 | 0.983-1.078 | 1.23 | 0.219 | 0.0% | 0.900 |
| Asian | A | 2 | 5791/5817 | 1.135 | 0.829-1.556 | 0.79 | 0.430 | 80.9% | 0.022 |
| Overall | A | 4 | 15351/15486 | 1.026 | 0.986-1.068 | 1.28 | 0.202 | 43.7% | 0.149 |
| **Major mood disorders** | | | | | | | | | |
| Caucasian | A | 9 | 19090/21802, 376 | 1.007 | 0.975-1.039 | 0.42 | 0.674 | 26.3% | 0.210 |
| Asian | A | 3 | 6266/6405 | 1.009 | 0.784-1.299 | 0.07 | 0.942 | 78.6% | 0.009 |
| Overall | A | 12 | 25356/28207, 376 | 0.991 | 0.939-1.046 | 0.32 | 0.747 | 45.8% | 0.042 |

**Table S13 Statistics of meta-analyses for rs947267 with BD, MDD and major mood disorders**

| **Disorder** | **Risk allele** | ***N* of comparisons** | ***N* of case/control,**  ***N* of families** | **Meta-analysis** | | | | **Heterogeneity** | |
| --- | --- | --- | --- | --- | --- | --- | --- | --- | --- |
|  |  |  |  | OR | 95% CI | *Z*-value | *P*-value | *I^2^* | *P*-value |
| **BD** | | | | | | | | | |
| Caucasian | C | 7 | 8911/11223, 376 | 1.014 | 0.973-1.057 | 0.65 | 0.513 | 10.7% | 0.348 |
| **MDD** | | | | | | | | | |
| Caucasian | C | 1 | 57/188 | 1.188 | 0.777-1.817 | 0.79 | 0.427 | N.A. | N.A. |
| Asian | C | 2 | 5791/5817 | 1.014 | 0.962-1.068 | 0.51 | 0.613 | 0.0% | 0.936 |
| Overall | C | 3 | 5848/6005 | 1.016 | 0.964-1.070 | 0.60 | 0.549 | 0.0% | 0.766 |
| **Major mood disorders** | | | | | | | | | |
| Caucasian | C | 9^1^ | 8968/11411, 1022 | 1.020 | 0.979-1.062 | 0.94 | 0.350 | 0.0% | 0.450 |
| Asian | C | 2 | 5791/5817 | 1.014 | 0.962-1.068 | 0.51 | 0.613 | 0.0% | 0.936 |
| Overall | C | 11 | 14759/17228, 1022 | 1.017 | 0.985-1.050 | 1.05 | 0.294 | 0.0% | 0.641 |

^1^In Gomez *et al.* study, MDD and BPD samples were combined together as “major mood disorders” group [17].

**Table S14 Statistics of meta-analyses for rs1935062 with BD, MDD and major mood disorders**

| **Disorder** | **Risk allele** | ***N* of comparisons** | ***N* of case/control,**  ***N* of families** | **Meta-analysis** | | | | **Heterogeneity** | |
| --- | --- | --- | --- | --- | --- | --- | --- | --- | --- |
|  |  |  |  | **OR** | **95% CI** | ***Z*-value** | ***P*-value** | ***I^2^*** | ***P*-value** |
| **BD** | | | | | | | | | |
| Caucasian | C | 8 | 2216/3453, 376 | 1.003 | 0.924-1.089 | 0.07 | 0.947 | 38.6% | 0.122 |
| **MDD** | | | | | | | | | |
| Caucasian | C | 1 | 57/188 | 1.247 | 0.817-1.904 | 1.02 | 0.306 | N.A. | N.A. |
| Asian | C | 1 | 5303/5337 | 0.982 | 0.930-1.037 | 0.65 | 0.517 | N.A. | N.A. |
| Overall | C | 2 | 5360/5525 | 0.986 | 0.934-1.041 | 0.51 | 0.609 | 17.0% | 0.272 |
| **Major mood disorders** | | | | | | | | | |
| Caucasian | C | 9 | 2273/3641, 376 | 1.011 | 0.933-1.096 | 0.26 | 0.794 | 35.4% | 0.135 |
| Asian | C | 1 | 5303/5337 | 0.982 | 0.930-1.037 | 0.65 | 0.517 | N.A. | N.A. |
| Overall | C | 10 | 7576/8978, 376 | 0.991 | 0.947-1.037 | 0.39 | 0.697 | 29.3% | 0.175 |


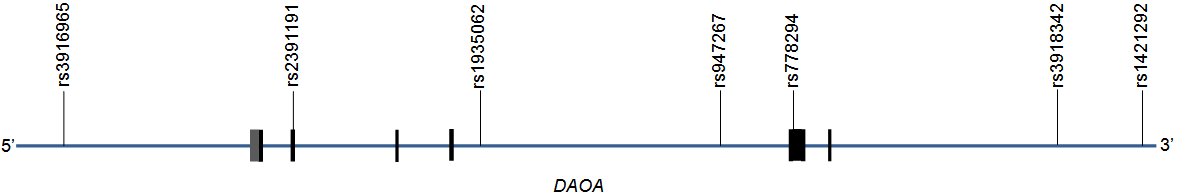


**Fig. S1 Schematic representation of the *DAOA* gene and the location of tested SNPs**

Exon is represented by black vertical lines, and UTR is indicated by gray lines.


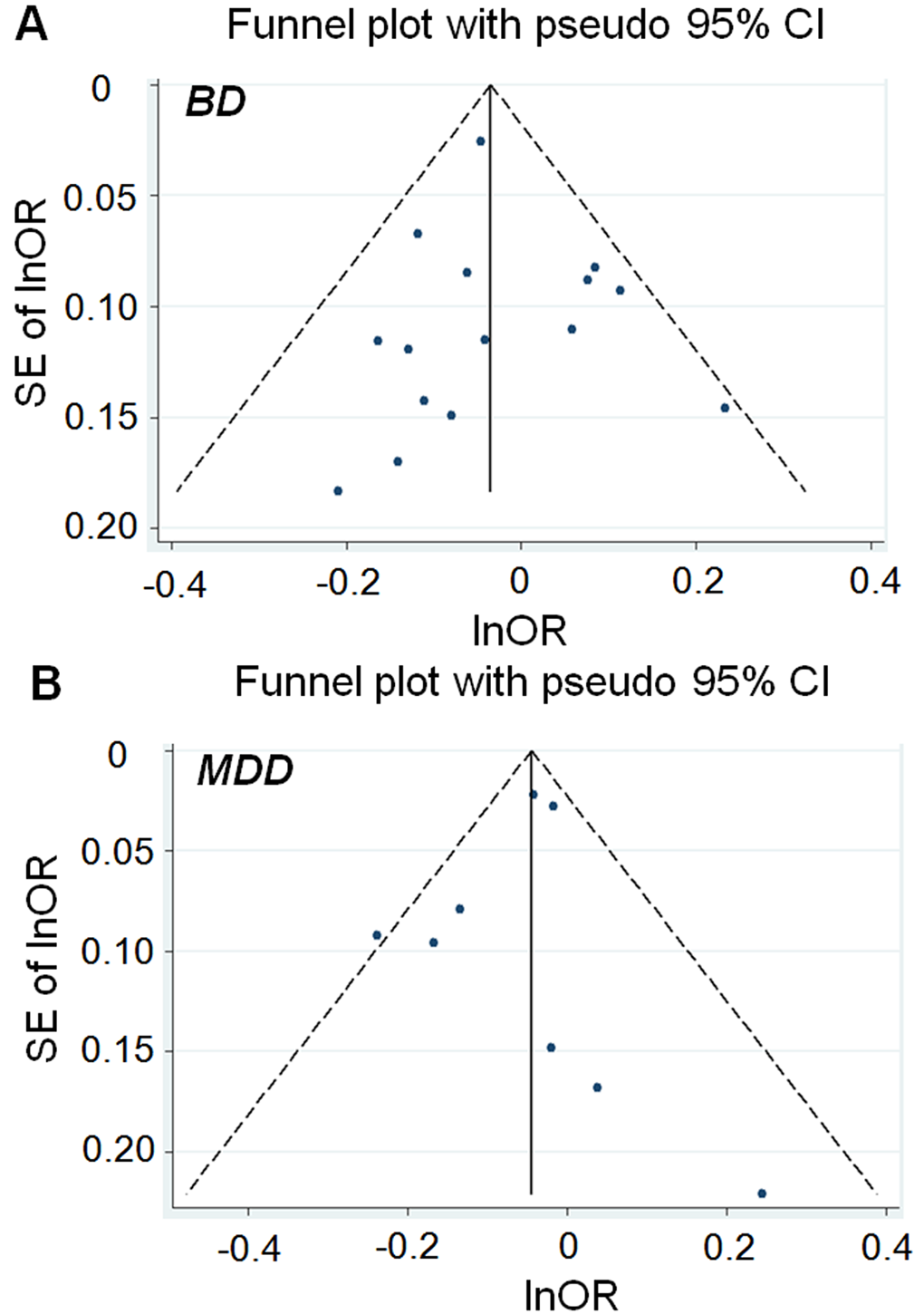


**Fig. S2 Begg’s funnel plot with pseudo 95% confidence limits for meta-analysis of rs2391191**

**
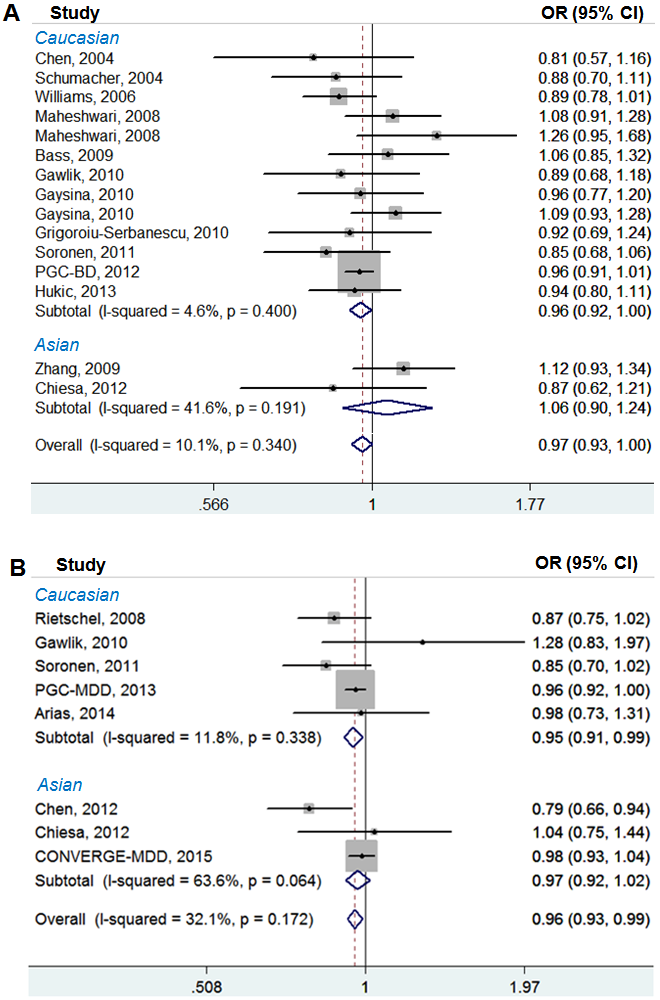
**

**Fig. S3 Forest plot of meta-analysis for allele-A rs2391191 in BD (A) and MDD (B) using the fixed-effect model**

Note: Overall refers to the combined samples of Caucasian and Asian populations.

**
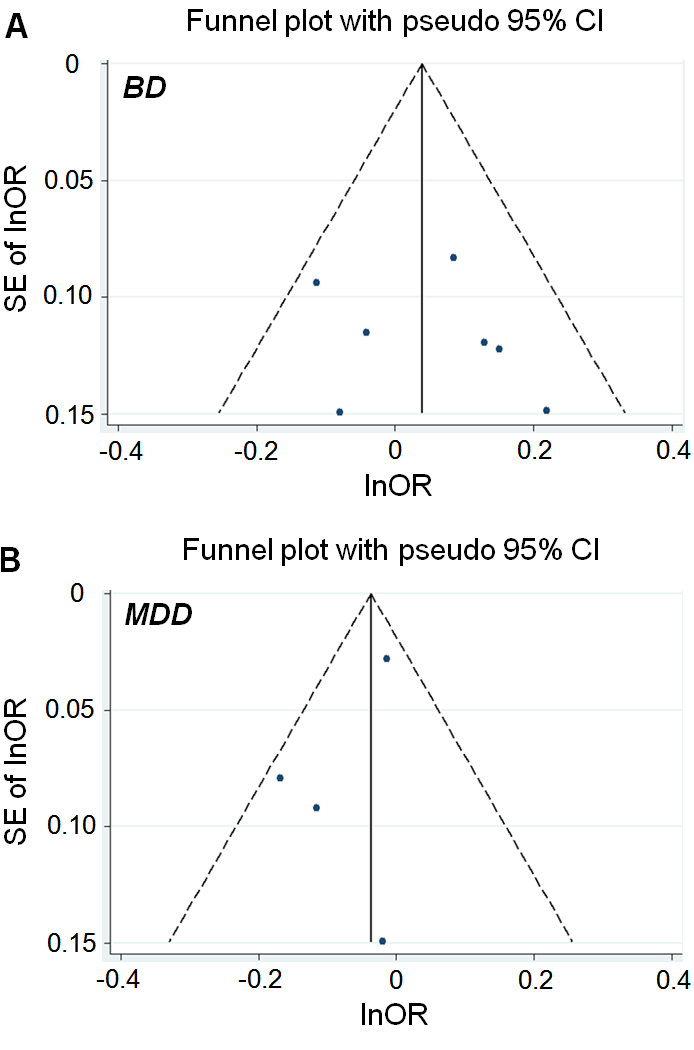
**

**Fig. S4 Begg’s funnel plot with pseudo 95% confidence limits for meta-analysis of rs3916965**


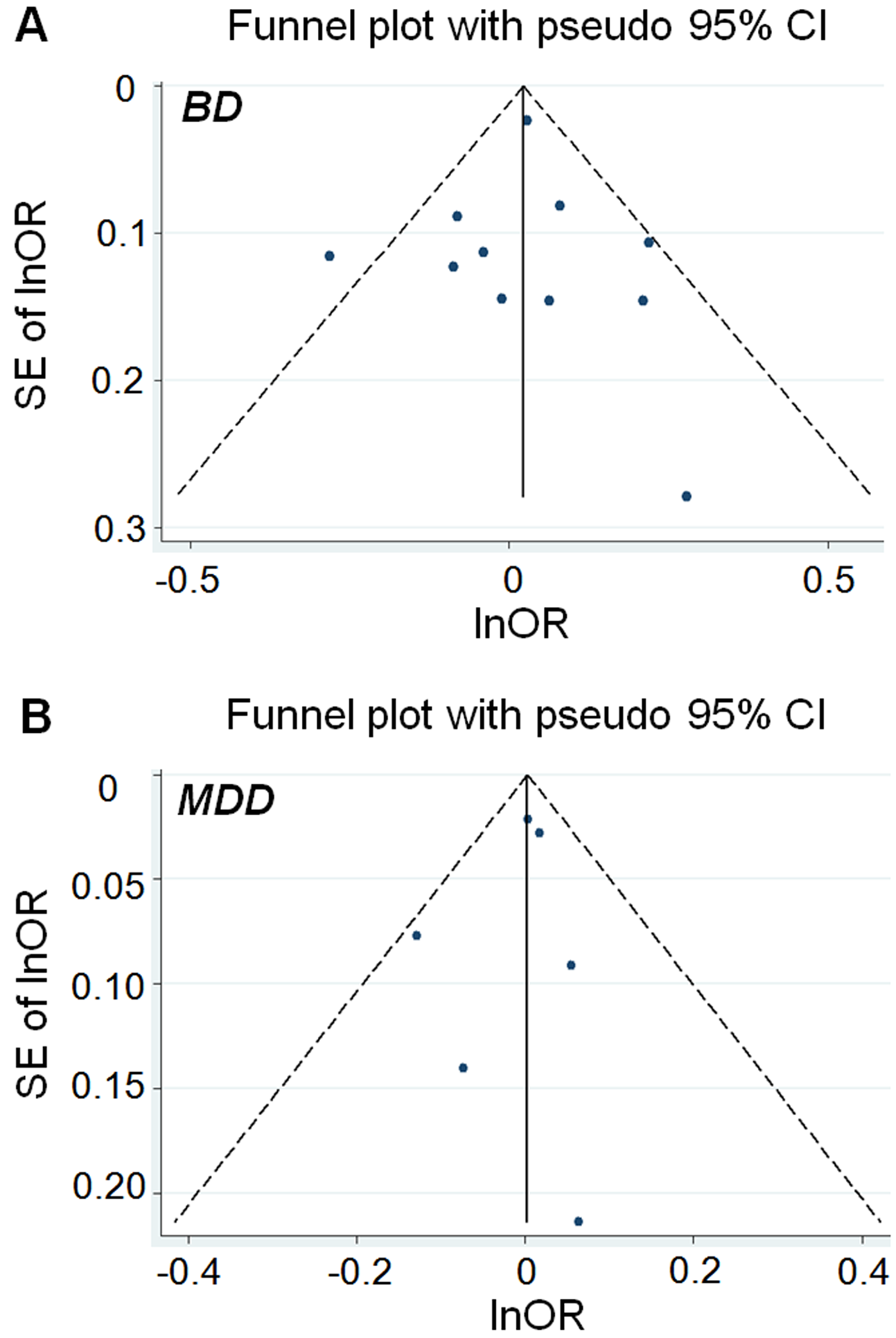


**Fig. S5 Begg’s funnel plot with pseudo 95% confidence limits for meta-analysis of rs3918342**


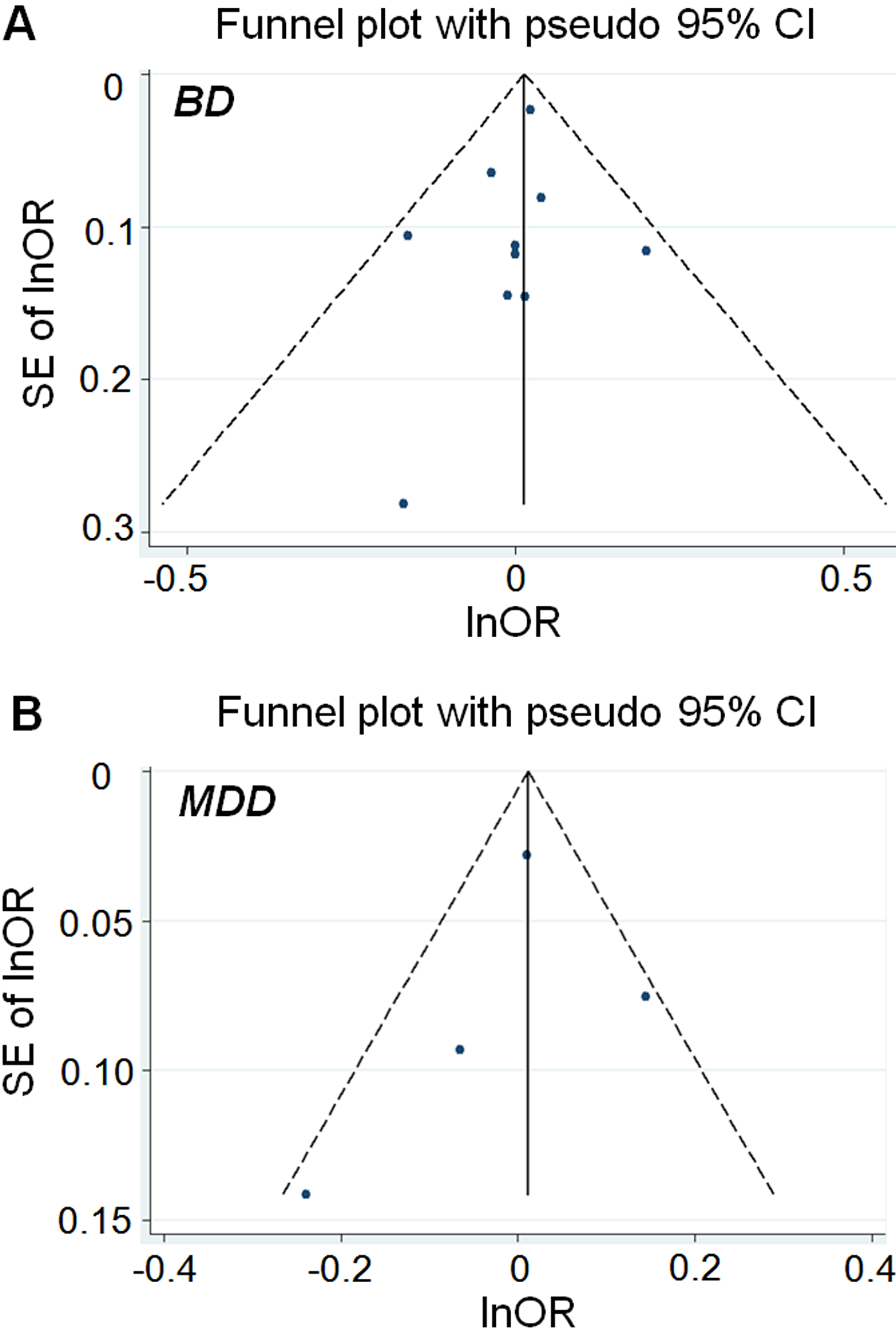


**Fig. S6 Begg’s funnel plot with pseudo 95% confidence limits for meta-analysis of rs1421292**


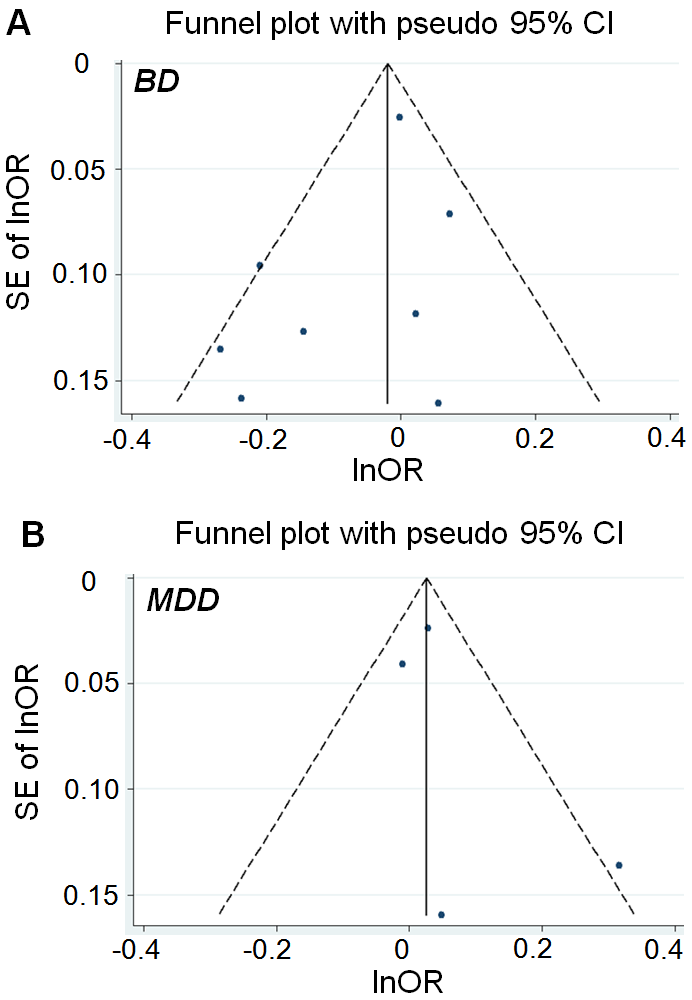


**Fig. S7 Begg’s funnel plot with pseudo 95% confidence limits for meta-analysis of rs778294**

**
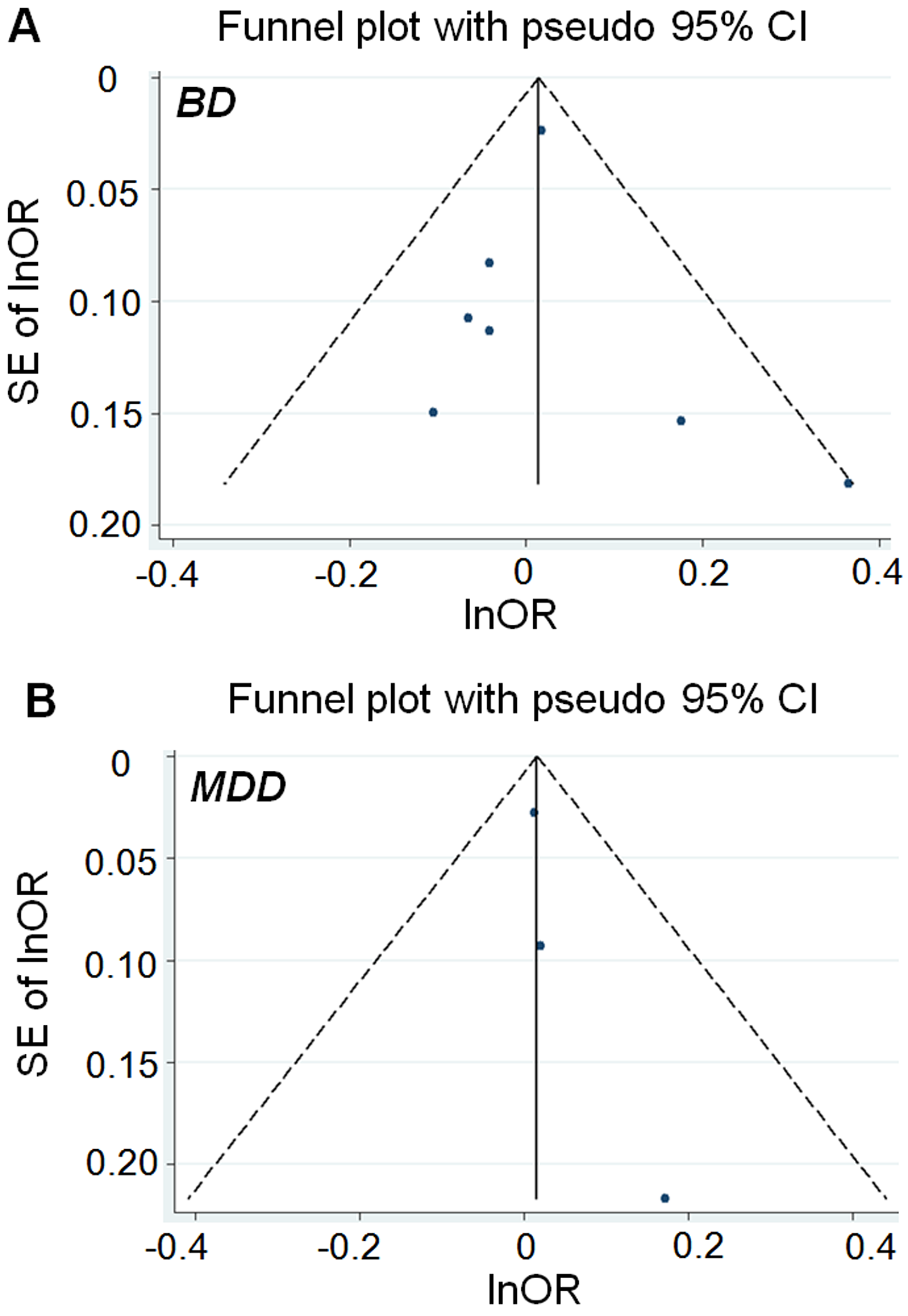
**

**Fig. S8 Begg’s funnel plot with pseudo 95% confidence limits for meta-analysis of rs947267**


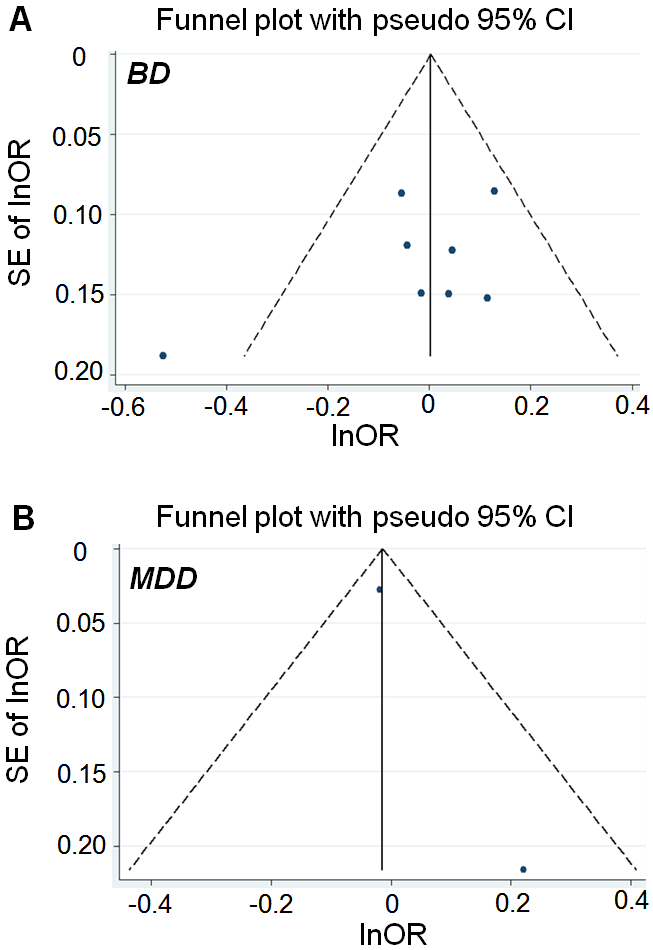


**Fig. S9 Begg’s funnel plot with pseudo 95% confidence limits for meta-analysis of rs1935062**


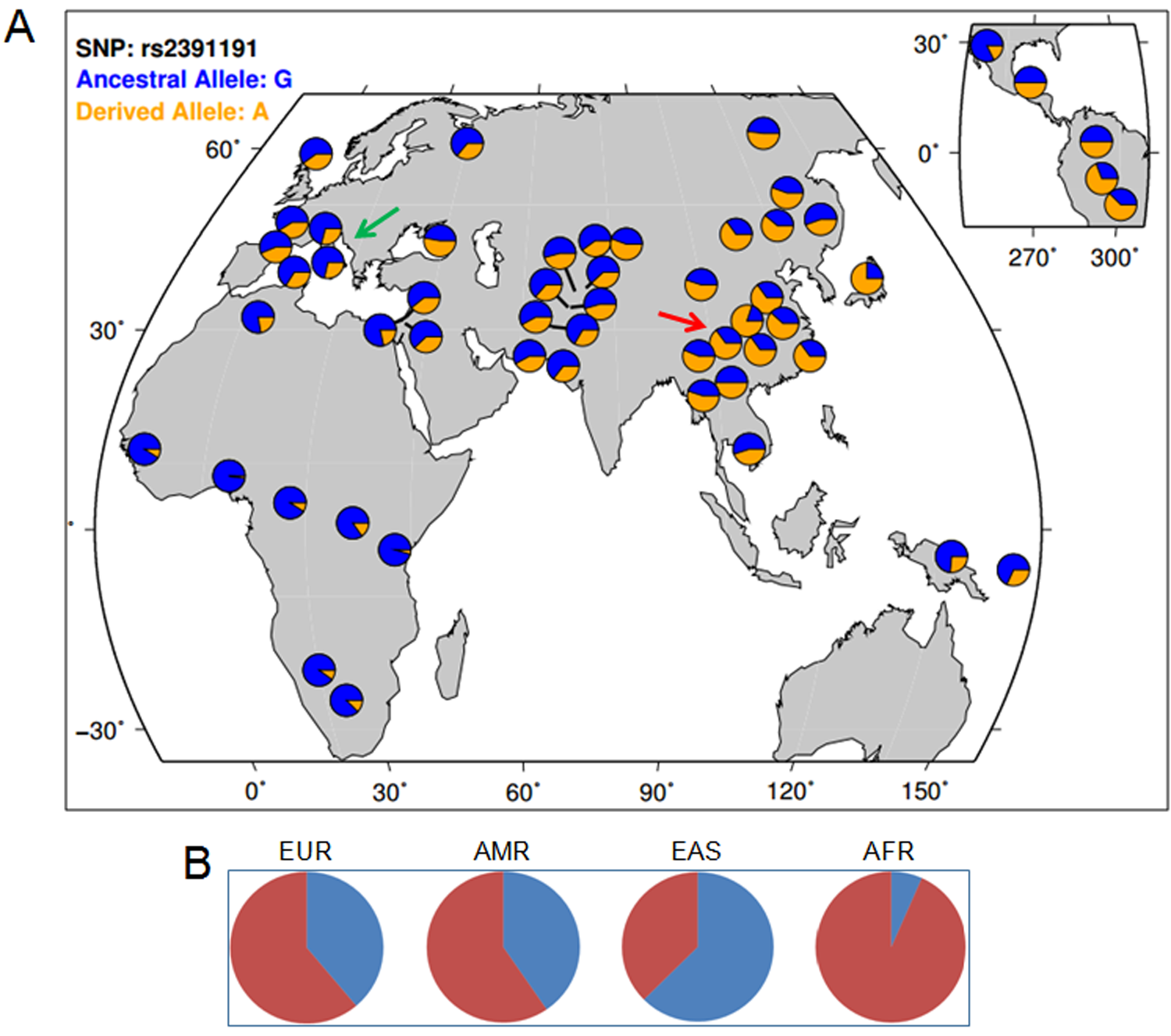


**Fig. S10** **Allele frequency distributions of rs2391191 in global populations**

(A) The risk allele (A-allele) of rs2391191 showed dramatic frequency differences among 53 world populations. The map was generated from HGDP selection browser (<http://hgdp.uchicago.edu/cgi-bin/gbrowse/HGDP/>) [37].

(B) Allele frequency distributions of rs2391191 in 4 main populations from the 1000 Genomes Projects (www.1000genomes.org). EUR, European; AMR, American; EAS, East Asian; AFR, African.

Supplementary references

1. Psychiatric GCBDWG. Large-scale genome-wide association analysis of bipolar disorder identifies a new susceptibility locus near ODZ4. *Nat Genet* 2011; 43: 977-983.

2. Ripke S, Wray NR, Lewis CM, Hamilton SP, Weissman MM, Breen G, et al. A mega-analysis of genome-wide association studies for major depressive disorder. *Mol Psychiatry* 2013; 18: 497-511.

3. CONVERGE Consortium. Sparse whole-genome sequencing identifies two loci for major depressive disorder. *Nature* 2015; 523: 588-591.

4. Ramasamy A, Trabzuni D, Guelfi S, Varghese V, Smith C, Walker R, et al. Genetic variability in the regulation of gene expression in ten regions of the human brain. *Nat Neurosci* 2014; 17: 1418-1428.

5. Maheshwari M, Shi J, Badner JA, Skol A, Willour VL, Muzny DM, et al. Common and rare variants of DAOA in bipolar disorder. *Am J Med Genet B Neuropsychiatr Genet* 2009; 150B: 960-966.

6. Grigoroiu-Serbanescu M, Herms S, Diaconu CC, Jamra RA, Meier S, Bleotu C, et al. Possible association of different G72/G30 SNPs with mood episodes and persecutory delusions in bipolar I Romanian patients. *Progress in neuro-psychopharmacology & biological psychiatry* 2010; 34: 657-663.

7. Schumacher J, Jamra RA, Freudenberg J, Becker T, Ohlraun S, Otte AC, et al. Examination of G72 and D-amino-acid oxidase as genetic risk factors for schizophrenia and bipolar affective disorder. *Molecular psychiatry* 2004; 9: 203-207.

8. Schulze TG, Ohlraun S, Czerski PM, Schumacher J, Kassem L, Deschner M, et al. Genotype-phenotype studies in bipolar disorder showing association between the DAOA/G30 locus and persecutory delusions: a first step toward a molecular genetic classification of psychiatric phenotypes. *Am J Psychiatry* 2005; 162: 2101-2108.

9. Zhang Z, Li Y, Zhao Q, Huang K, Wang P, Yang P, et al. First evidence of association between G72 and bipolar disorder in the Chinese Han population. *Psychiatr Genet* 2009; 19: 151-153.

10. Arias B, Fabbri C, Serretti A, Drago A, Mitjans M, Gasto C, et al. DISC1-TSNAX and DAOA genes in major depression and citalopram efficacy. *Journal of affective disorders* 2014; 168: 91-97.

11. Rietschel M, Beckmann L, Strohmaier J, Georgi A, Karpushova A, Schirmbeck F, et al. in large population-based groups from Germany. *Am J Psychiatry* 2008; 165: 753-762.

12. Chen J, Xu Y, Zhang J, Liu Z, Xu C, Zhang K, et al. Genotypic association of the DAOA gene with resting-state brain activity in major depression. *Mol Neurobiol* 2012; 46: 361-373.

13. Bass NJ, Datta SR, McQuillin A, Puri V, Choudhury K, Thirumalai S, et al. Evidence for the association of the DAOA (G72) gene with schizophrenia and bipolar disorder but not for the association of the DAO gene with schizophrenia. *Behav Brain Funct* 2009; 5: 28.

14. Gawlik M, Wehner I, Mende M, Jung S, Pfuhlmann B, Knapp M, et al. The DAOA/G30 locus and affective disorders: haplotype based association study in a polydiagnostic approach. *BMC Psychiatry* 2010; 10: 59.

15. Gomez L, Wigg K, Feng Y, Kiss E, Kapornai K, Tamas Z, et al. *ed Genet B Neuropsychiatr Genet* 2009; 150B: 1007-1012.

16. Zuliani R, Moorhead TW, Job D, McKirdy J, Sussmann JE, Johnstone EC, et al. Genetic variation in the G72 (DAOA) gene affects temporal lobe and amygdala structure in subjects affected by bipolar disorder. *Bipolar Disord* 2009; 11: 621-627.

17. Psychiatric, G.C.B.D.W.G., Large-scale genome-wide association analysis of bipolar disorder identifies a new susceptibility locus near ODZ4. Nat Genet, 2011. 43(10): p. 977-83.

18. Major Depressive Disorder Working Group of the Psychiatric, G.C., et al., A mega-analysis of genome-wide association studies for major depressive disorder. Mol Psychiatry, 2013. 18(4): p. 497-511.

19. Williams NM, Green EK, Macgregor S, Dwyer S, Norton N, Williams H, et al. Variation at the DAOA/G30 locus influences susceptibility to major mood episodes but not psychosis in schizophrenia and bipolar disorder. *Arch Gen Psychiatry* 2006; 63: 366-373.

20. Chen YS, Akula N, Detera-Wadleigh SD, Schulze TG, Thomas J, Potash JB, et al. Findings in an independent sample support an association between bipolar affective disorder and the G72/G30 locus on chromosome 13q33. *Mol Psychiatry* 2004; 9: 87-92.

21. Hukic DS, Frisen L, Backlund L, Lavebratt C, Landen M, Traskman-Bendz L, et al. Cognitive manic symptoms in bipolar disorder associated with polymorphisms in the DAOA and COMT genes. *PLoS One* 2013; 8: e67450.

.
