# Supplementary material for: Allelic frequency differences of DAOA variants between Caucasians and Asians and their association with major mood disorders
Source: Signal Transduct Target Ther. 2019 Oct 4;4:39. doi: 10.1038/s41392-019-0066-5 (PMC6799844; doi:10.1038/s41392-019-0066-5)
Supplement: Supplementary file 4 — Table S1 PDF [file 41392_2019_66_MOESM4_ESM.pdf]

Table S1 Characteristics of the studies included in the meta-analysis

BD, bipolar disorder; MDD, major depressive disorder; FP-TDI, template-directed dyeterminator inc

For GWAS, the detailed description of sample information and genotyping method can be found in

Author	Publicaito n year	Region	Ethnicity	Disorder	Definition
**GWASs**					
PGC-BD	2012	Multiple	Caucasian	BD	DSM-IIR/IV, RDC
PGC-MDD	2013	Multiple	Caucasian	MDD	DSM-IV
CONVERGE-MDD	2015	China	Asian	MDD	DSM-IV
**Candidate gene studies**					
Chen *et al.*	2004	Maryland and Iowa	Caucasian	BD	Schedule for Affectiv
Schumacher *et al.*	2004	Germany	Caucasian	BD	DSM-IV
Schulze *et al.*	2005	Germany	Caucasian	BD	DSM-IV
		Poland	Caucasian	BD	SCID
Williams *et al.*	2006	U.K.	Caucasian	BD	DSM-IV
Gomez *et al.*	2008	Hungary	Caucasian	Mood disorde	DSM-IV
Maheshwari *et al.*	2008	USA	Caucasian	BD	DSM-III-R or DSM-IV
		USA	Caucasian	BD	DSM-III-R or DSM-IV
Rietschel *et al.*	2008	Germany	Caucasian	MDD	DSM-IV
Bass et al.	2009	U.K.	Caucasian	BD	SADS-L
Zhang *et al.*	2009	China	Asian	BD	DSM
Zuliani *et al.*	2009	U.K.	Caucasian	BD	DSM-IV
Gawlik et al.	2010	German	Caucasian	BD	ICD-10
		German	Caucasian	MDD	ICD-10
Gaysina *et al.*	2010	Canada	Caucasian	BD	DSM-IV
		U.K.	Caucasian	BD	DSM-IV
Grigoroiu-Serbane	2010	Romania	Caucasian	BD	DSM-IV
Soronen *et al.*	2011	Finland	Caucasian	BD	DSM-IV
		Finland	Caucasian	MDD	DSM-IV
Chen *et al.*	2012	China	Asian	MDD	DSM-IV
Chiesa *et al.*	2012	Korea	Asian	BD	DSM-IV
		Korea	Asian	MDD	DSM-IV
Hukic *et al.*	2013	Sweden	Caucasian	BD	DSM-IV
Arias *et al.*	2014	Spain	Caucasian	MDD	DSM-IV

:orporation with fluorescence polarization detection.
original manuscript.

Genotyping method	Cases			Controls			Title
	N	Age (years)	Male (%)	Controls	Age (years)	Male (%)	
Multiple platforms	7481	N.A.	N.A.	9250	N.A.	N.A.	Large-scale
Multiple platforms	9240	N.A.	N.A.	9519	N.A.	N.A.	A mega-an
Genome sequencing	5303	N.A.	N.A.	5337	N.A.	N.A.	Sparse who
FP-TDI; PCR-RFLP	139	N.A.	N.A.	113	N.A.	N.A.	Findings in
Masscode Technology	300	42.3 (13.2)	138 (46%)	300	47.1 (15.2)	121 (40.3)	Examinatio
Masscode Technology	300	42.3 (13.2)	138 (46.0)	300	47.1 (15.2)	121 (40.3)	Genotype-I
Sequenom	294	46.9 (13.7)	128 (43.5)	311	43.7 (9.5)	115 (37.0)	
Allele-specific PCR; sing	706	41.8 (13.5)	503 (70.9)	1416	42.4 (11.1)	731 (51.6)	Variation a
TaqMan	646 nuclear	N.A.	N.A.		N.A.	N.A.	G72/G30 (I
Sanger sequencing	555	N.A.	228 (41.1)	564	N.A.	282 (50.0)	Common a
Illumina	376 nuclear	families (157 males)					
Sequenom	500	47.9 (13.8)	178 (35.6)	1030	47.9 (15.5)	499 (48.4)	G72 and Its
KASPar SNP genotyping	303	N.A.	N.A.	433	N.A.	N.A.	Evidence fo
Allelic specific multiple li	475	N.A.	255 (53.7)	588	N.A.	178 (30.3)	First evider
TaqMan	38	N.A.	19 (50.0)	81	N.A.	42 (51.9)	Genetic var
TaqMan	191	N.A.	N.A.	188	30.2 (10.7)	105 (59.0)	The DAOA
TaqMan	57	N.A.	N.A.	188	30.2 (10.7)	105 (59.0)	
SNPlex Genotyping Syst	385	46.0 (12.5)	142 (36.9%	312	43.7 (13.1)	144 (46.2)	Association
SNPlex Genotyping Syst	515	48.0 (11.4)	180 (35.0%	1316	41.7 (13.2)	553 (42.0)	
Sequenom	198	41.11 (13.48	84 (42.1)	180	41.29 (12.3	74 (40.0)	Possible as
Sequenom	178	N.A.	N.A.	1322	N.A.	562 (42.5)	P2RX7 Ger
Sequenom	272	N.A.	N.A.	1322	N.A.	562 (42.5)	
Sanger sequencing	488	37.6 (10.3)	209 (42.8)	480	36.8 (11.0)	214 (44.6)	Genotypic
Sanger sequencing	132	36.4 (11.6)	87 (65.9)	170	38.8 (12.8)	105 (61.8)	DAOA Vari
Sanger sequencing	145	41.4 (14.1)	75 (51.7)	170	38.8 (12.8)	105 (61.8)	
TaqMan	488	>18	209(43%)	1044	>18	616(59%)	Cognitive N
TaqMan	320	N.A.	N.A.	150	N.A.	N.A.	DISC1-TSN

Phenotype Studies in Bipolar Disorder Showing Association Between the DAOA/G30 Locus and Perse

cutory Delusions: A First Step Toward a Molecular Genetic Classification of Psychiatric Phenotypes
